# Prognostic Factors for Cognitive Recovery Beyond Early Poststroke Cognitive Impairment (PSCI): A Prospective Cohort Study of Spontaneous Intracerebral Hemorrhage

**DOI:** 10.3389/fneur.2020.00278

**Published:** 2020-04-28

**Authors:** Li Gong, Yongzhe Gu, Qiuyue Yu, Haichao Wang, Xiaoping Zhu, Qiong Dong, Rong Xu, Yanxin Zhao, Xueyuan Liu

**Affiliations:** ^1^Department of Neurology, Shanghai Tenth People's Hospital, Tongji University, Shanghai, China; ^2^School of Nursing, Second Military Medical University, Shanghai, China; ^3^Department of Nursing, Shanghai Tenth People's Hospital, Tongji University, Shanghai, China; ^4^Department of Neurosurgery, Huashan Hospital, Fudan University, Shanghai, China

**Keywords:** intracerebral hemorrhage, stroke, dementia, poststroke cognitive impairment, vascular cognitive impairment, red blood cell, cohort study

## Abstract

**Background:** Poststroke cognitive impairment (PSCI) has been increasingly recognized in patients, but some stroke survivors appear to show cognitive improvement beyond the acute stage. The risk factors associated with cognitive recovery after spontaneous intracerebral hemorrhage (ICH) onset have not yet been sufficiently investigated in prospective studies.

**Objective:** We aimed to identify the trajectory of post-ICH cognitive impairment and the association of potential prognostic factors with follow-up cognitive recovery beyond early PSCI.

**Methods:** In this stroke center-based cohort study, 141 consecutive dementia-free patients with spontaneous ICH were included and underwent Montreal Cognitive Assessment (MoCA) evaluation for cognitive function at baseline (within 2 weeks of ICH onset) and the shortened MoCA (short-MoCA) at a 6-month follow-up. To explore the prognostic factors associated with trajectory of cognition after an ICH onset, we adjusted for demographic and vascular risk factors, using multivariate logistic regression analysis.

**Results:** Of the 141 ICH patients, approximately three quarters (106/141) were diagnosed with early PSCI (MoCA score <26) within 2 weeks of ICH onset. The multiple logistic regression indicated independent positive associations between risk of early PSCI and dominant-hemisphere hemorrhage [odd's ratio (OR): 8.845 (3.347–23.371); *P* < 0.001], mean corpuscular volume (MCV) [OR: 1.079 (1.002–1.162); *P* = 0.043], admission systolic blood pressure (sBP) [OR: 1.021 (1.005–1.038); *P* = 0.012]. Furthermore, 36% (33/90) of ICH survivors who had early PSCI exhibited cognitive recovery at the 6-month follow-up. After examining potential predictors through multiple linear regression based on stepwise, there were independent negative associations between cognitive recovery and dominant hemisphere hemorrhage [OR: 6.955 (1.604–30.162); *P* < 0.01], lobar ICH [OR: 8.363 (1.479–47.290); *P* = 0.016], years of education ≤ 9 [OR: 5.145 (1.254–21.105); *P* = 0.023], and MCV [OR: 1.660 (1.171–2.354); *P* = 0.004]. Baseline cognitive performance in the domains of visuospatial/executive function, attention, orientation, and language showed positive correlations with cognitive improvement (*P* < 0.05).

**Conclusion:** In this cohort study of dementia-free survivors of ICH, our results show that one in three early PSCI survivors exhibit cognitive recovery, in relation to dominant-hemisphere hematoma, lobar ICH, educational history, and MCV levels. Future clinical trials including ICH survivors with cognitive dysfunction should assess these factors.

## Introduction

Accounting for 20% of all strokes, intracerebral hemorrhage (ICH) is a major contributor to physical disability and mortality, although its association with cognitive impairment is not well-established (1, 2). In contrast to the increasingly recognized poststroke cognitive impairment (PSCI), which is increasingly recognized as associated with ischemic stroke (3), the incidence of cognitive dysfunction and the risk factors associated with the cognitive trajectory after ICH onset remain mostly unknown. This is likely due to the lower incidence of ICH and the high mortality rate observed during the acute stage (4–6). Indeed, ICH survivors are at high risk of cognitive decline following stroke onset, consequently affecting quality of life, and posing an increased social and economic burden. Although most previous studies have focused on cognitive decline, certain stroke patients have shown cognitive improvement beyond the acute stage (7–9). In particular, there is a lack of information about whether there are potential prognostic factors for the recovery of cognitive function 6 months after ICH onset.

Our recently published results and previous findings suggest that abnormal red blood cell (RBC) indices are correlated with poor outcomes in survivors with acute stroke or cognitive dysfunction after aneurysmal subarachnoid hemorrhage (aSAH) (10–12). Therefore, we hypothesized that RBC indices might be related to cognitive functioning after ICH onset. To the best of our knowledge, no prospective study has ever investigated cognitive recovery over time in ICH patients with immediate PSCI. We sought to determine prognostic factors for cognitive recovery in a prospective cohort of ICH patients over a 6-month follow-up period. We used two separate analyses to investigate the risk of developing cognitive impairment within 2 weeks of ICH onset and identify prognostic factors for cognitive recovery 6 months after ICH onset.

## Methods

### Study Design and Subjects

In order to investigate the prognostic factors for cognitive trajectory post-ICH, we designed a two-stage scheme, including separate evaluations for early PSCI (within 2 weeks of ICH onset) and predictors for cognitive recovery (beyond 6 months of ICH onset). At the first stage, we recruited patients (age ≥18 years) hospitalized in our stroke center from July 2017 to December 2018 who were diagnosed with spontaneous ICH based on computed tomography (CT) scan within 2 weeks of ICH onset. ICH survivors were analyzed within 2 weeks of ICH onset to evaluate early PSCI. During the next stage, we only included ICH survivors with early PSCI to investigate the prognostic factors for cognitive trajectory, particularly cognitive recovery. The exclusion criteria were as follows: (1) the hemorrhagic stroke was related to brain trauma/tumor, hemorrhagic transformation of an ischemic stroke, or rupture of a vascular malformation/aneurysm; (2) inability to participate in cognitive assessment due to prominent aphasia, deafness, unstable consciousness defined as Glasgow Coma Scale (GCS) <14 scores, or difficulty in completing the procedures of the study; and (3) history of psychiatric disorders or diagnosis of cognitive impairment via existing medical records in order to limit the effect of preexisting cognitive status on current cognitive function. This study was approved by the Ethics Committee of Shanghai Tenth People's Hospital (Shanghai, China) and was conducted in accordance with the Declaration of Helsinki. All participants or their legal guardians (spouse or child) provided informed consent.

### Evaluations of Baseline Cognitive Function and Neuroimaging

Baseline cognitive function was assessed using the Chinese version of the Montreal Cognitive Assessment (MoCA), with scores ranging from 0 (worst performance) to 30 (best performance). The MoCA scale has been validated as an effective and sensitive assessment method for the identification of mild cognition impairment (13). In this study, cognitive impairment in the acute stage was defined as a MoCA score <26, according to a previously published recommendation (14). We applied the MoCA correction with an addition of one point when patients had <12 years of education. Considering the risk of confusion and delirium due to ICH and perihematoma edema of hemorrhage during the very-early period, cognitive evaluations were performed on the day of discharge (most of these took place 12–14 days after ICH onset), as the edema decreased and the consciousness was stable (GCS ≥14 scores).

To quantify the severity of white matter hyperintensity (WMH) and perihematoma lesions, we performed a magnetic resonance imaging assessment, which included a fluid-attenuated inversion recovery (FLAIR) sequence and a diffusion-weighted imaging sequence (Magnetom Verio, Siemens). WMH was defined as a focal lesion in the white matter with corresponding hyperintensity on the FLAIR sequence. We assessed the presence and severity of WMHs, and deep and periventricular WMHs were graded according to the Fazekas scale (grades 0–3) (15). Moreover, we evaluated patients for intracranial stenosis using CT angiography or magnetic resonance angiography (MRA) and defined intracranial stenosis as >50% diameter stenosis, as previously described (16). The anatomical locations (lobar/non-lobar) and sizes (volume) of the hematomas were included in the hospital-based information. All neuroimaging parameters were evaluated by two experienced investigators (C.W.L and Z.R.L) who were blinded to the clinical data.

### Six-Month Follow-Up Cognitive Evaluation

ICH survivors and/or their caregivers were followed up 6 months after the ICH. At the visit, cognitive evaluation was performed using the shortened MoCA (short-MoCA) at 6 months, and the investigators were blind to both the previous evaluation and laboratory results. The short-MoCA has been validated as an optimal brief screening tool for cognitive impairment rehabilitation patients (17). It includes a six-item orientation task, a one-item phonemic fluency test, and a five-word immediate and delayed memory test with scores ranging from 0 (worst performance) to 12 (best performance). This made it possible to be administered by telephone and reduce lost rate of follow-up. In the present study, cognitive impairment at the follow-up was defined as a short-MoCA score <7, according to a previously published recommendation (18). We utilized the date of the last short-MoCA as the end point of the follow-up, and only cognitive assessments occurring before this end point were included in the study. All data were censored in case of death or loss to follow-up.

### Statistical Analysis

The Kolmogorov–Smirnov test and the Levene test were performed to explore the normality and homogeneity of quantitative variables, respectively. Quantitative variables with normal distribution were expressed as mean ± standard deviation, quantitative variables with abnormal distribution as quartiles, and qualitative variables as frequencies with rate. We then used univariate analysis to evaluate the association between each variable and cognitive performances, at both acute and follow-up stages Furthermore, potential predictors of cognition were examined through analysis of multicollinearity by stepwise regression. Finally, binary logistic regression analysis was employed to investigate the independent predictors for cognitive recovery. The backward LR method was used to evaluate the strength according to the odds ratio (OR) and corresponding 95% confidence interval (CI), with the *F* probability of entry set at 0.05 and that of removal set at 0.10. All statistical analyses were performed using SPSS software, version 22.0 (SPSS Inc., USA), and a *P* < 0.05 was considered statistically significant.

## Results

### Baseline Characteristics in Relation to Cognitive Impairment Within 2 Weeks

Of the 141 participants with primary ICH included in this study, 106 (75.2%) patients were diagnosed with cognitive impairment at the acute stage (MoCA <26). As shown in Table 1, the univariate analysis showed that the ICH patients with early PSCI were older, exhibited a dominant-hemisphere hemorrhage, had a higher admission systolic blood pressure (sBP), aspartate aminotransferase (AST) level, ratio of aspartate aminotransferase to alanine aminotransferase (AST/ALT), and had greater severity of white matter hyperintensities and constipation. After adjusting for factors significant in the univariate analysis, such as age, sBP, hemorrhage side, and RBC indices, we found that dominant-hemisphere hemorrhage [OR: 8.845 (3.347–23.371); *P* < 0.001] and admission sBP [OR: 1.021 (1.005–1.038); *P* = 0.012] were independently associated with increased risks of early PSCI (Table 2). Moreover, our data showed that ICH survivors with abnormal RBC indices [in particular, high mean corpuscular volume (MCV) levels] were more likely to develop early PSCI [OR: 1.079 (1.002–1.162); *P* = 0.043].

**Table 1:** Baseline characteristics in relation to cognitive impairment in the acute stage (*n* = 141).

	**Total *n* = 141**	**Early PSCI *n* = 106**	**Non-PSCI *n* = 35**	**Univariable analysis**		**Multivariate analysis^g^**	
				**OR and 95% CI**	***P*-value**	**OR and 95% CI**	***P*-value**
**Demographic variables**							
Age, years	66.75 ± 12.86	68.16 ± 13.05	62.46 ± 11.38	1.036 (1.004–1.068)	**0.025**		
Male	90 (63.8%)	70 (66.0%)	20 (57.1%)	1.458 (0.668–3.184)	0.344		
Educational years ≤ 9	75 (53.2%)	61 (57.5%)	14 (40.0%)	2.033 (0.934–4.428)	0.074		
Baseline NIHSS scores	4 (2–6)	4 (2–6)	4 (2–5)	0.990 (0.901–1.088)	0.834		
sBP on admission, mmHg	161.92 ± 27.24	165.98 ± 26.09	149.60 ± 27.32	1.025 (1.008–1.041)	**0.003**	1.021 (1.005–1.038)	**0.012**
**History**							
Hypertension	125 (88.7%)	96 (90.6%)	29 (82.9%)	1.986 (0.665–5.931)	0.219		
Diabetes mellitus	49 (34.8%)	34 (32.1%)	15 (42.9%)	0.630 (0.288–1.379)	0.247		
Excessive smoking^a^	41 (29.1%)	30 (28.3%)	11 (31.4%)	0.861 (0.376–1.974)	0.724		
Excessive drinking^b^	35 (24.8%)	29 (27.4%)	6 (17.1%)	1.820 (0.685–4.837)	0.230		
**Complications in hospital**							
Fever^c^	29 (20.6%)	24 (22.6%)	5 (14.3%)	1.756 (0.614–5.020)	0.293		
Constipation^d^	36 (25.5%)	32 (30.2 %)	4 (11.4%)	3.351 (1.093–10.280)	**0.034**		
Hyperglycemia^e^	42 (29.8%)	31 (29.2%)	11 (31.4%)	0.902 (0.394–2.063)	0.807		
Hyponatremia^f^	44 (31.2%)	31 (29.2%)	13 (37.1%)	0.699 (0.313–1.562)	0.383		
**Imaging variables**							
ICH volume, ml	4.07 (1.24–11.06)	4.81 (1.24–11.97)	3.31 (0.66–7.13)	1.046 (0.993–1.103)	0.093		
Dominant-hemisphere hemorrhage	91 (64.5%)	79 (74.5%)	12 (34.3%)	5.608 (2.461–12.777)	**<0.001**	8.845 (3.347–23.371)	**<0.001**
Lobar hemorrhage	41 (29.1%)	32 (30.2%)	9 (25.7%)	1.249 (0.527–2.964)	0.614		
Intracranial arterial stenosis	45 (31.9%)	34 (32.1%)	11 (31.4%)	1.264 (0.528–3.029)	0.599		
without assessment	34 (24.1%)	28 (26.4%)	6 (17.1%)				
White matter hyperintensities	69 (48.9%)	64 (60.4%)	5 (14.3%)	24.000(6.870–83.838)	**<0.001**		
without assessment	49 (34.8%)	34 (32.1%)	15 (42.9%)				
**Laboratory variables**							
CRP, mg/L	3.36 (3.02–8.61)	3.32 (3.02–9.52)	3.36 (3.23–4.87)	1.013 (0.986–1.042)	0.345		
RBC, ×10^12^/L	4.48 ± 0.55	4.41 ± 0.53	4.70 ± 0.55	0.350 (0.163–0.752)	**0.007**	0.414 (0.154–1.114)	0.081
Hb, g/L	135.01 ± 17.20	133.87 ± 16.87	138.46 ± 17.95	0.985 (0.963–1.007)	0.172		
HCT, %	40.44 ± 4.61	40.16 ± 4.52	41.28 ± 4.83	0.948 (0.872–1.031)	0.213		
MCV, fL	90.80 ± 6.67	91.64 ± 6.09	88.23 ± 7.73	1.075 (1.016–1.138)	**0.013**	1.079 (1.002–1.162)	**0.043**
MCH, pg	30.31 ± 2.64	30.55 ± 2.51	29.59 ± 2.90	1.138 (0.991–1.307)	0.067		
MCHC, g/L	333.50 ± 10.98	333.02 ± 11.78	334.97 ± 8.03	0.984 (0.950–1.019)	0.362		
RDW, %	13.28 ± 0.89	13.32 ± 0.91	13.19 ± 0.83	1.175 (0.761–1.816)	0.467		
PRO, g/L	66.48 ± 4.76	65.97 ± 5.11	68.03 ± 3.08	0.897 (0.814–0.989)	**0.029**	–	–
ALT, U/L	18.11 ± 12.58	18.18 ± 13.73	17.91 ± 8.34	1.002 (0.971–1.033)	0.912		
AST, U/L	22.16 ± 16.82	23.62 ± 19.04	17.76 ± 4.39	1.148 (1.042–1.266)	**0.005**		
AST/ALT	1.39 ± 0.59	1.47 ± 0.60	1.14 ± 0.45	3.705 (1.531–8.966)	**0.004**	–	–
Cr	70.48 ± 22.20	68.85 ± 15.21	75.42 ± 35.80	0.988 (0.972–1.004)	0.143		
eGFR, ml/min	88.03 ± 14.90	88.27 ± 13.87	87.28 ± 17.85	1.004 (0.979–1.030)	0.733		
FBG, mmol/L	5.97 ± 1.19	5.98 ± 1.21	5.92 ± 1.14	1.043 (0.751–1.447)	0.802		
LDL, mmol/L	2.43 ± 0.79	2.38 ± 0.78	2.59 ± 0.82	0.717 (0.446–1.153)	0.170		
HCY, μmol/L	13.39 ± 6.40	13.86 ± 7.03	11.96 ± 3.67	1.092 (0.974–1.225)	0.131		

*Values in bold indicate significant difference*.

a *Excessive smoking was defined as a daily intake of at least one cigarette for more than 1 year*.

b *Excessive drinking was defined as a weekly intake of at least 200 g liquor for more than 1 year*.

c *Fever was defined as the body temperature above 38°C*.

d *Constipation was defined as defecation less than three times a week*.

e *Hyperglycemia was defined as the random blood glucose >11.1 mmol/L*.

f *Hyponatremia was defined as the serum sodium concentration <135 mmol/L*.

g *Multivariate analysis adjusted for age, sBP on admission, dominant-hemisphere, RBC, MCV, PRO, and AST/ALT*.

**Table 2:** Logistic regression model in relation to cognitive impairment in the acute stage (*n* = 141).

	***B***	**SE**	**Wald**	**OR**	**95% CI**	***P* value**
sBP on admission, mmHg	0.021	0.008	6.239	1.021	1.005–1.038	**0.012**
Dominant-hemisphere hemorrhage	2.180	0.496	19.334	8.845	3.347–23.371	**<0.001**
RBC, ×10^12^/L	−0.882	0.505	3.048	0.414	0.154–1.114	0.081
MCV, fL	0.076	0.038	4.099	1.079	1.002–1.162	**0.043**
Constant	−6.255	5.264	1.412	0.002		0.235

*Values in bold indicate significant difference*.

*Multivariate analysis adjusted for age, sBP on admission, dominant-hemisphere, RBC, MCV, PRO, and AST/ALT*.

### Six-Month Follow-Up Characteristics of the Subjects and Prognostic Factors of Cognitive Improvement

At the 6-month follow-up, besides 2 deaths and 14 losses to follow-up, 90 ICH survivors with early PSCI were finally included. Fifty-seven (63.3%) patients were diagnosed with cognitive impairment (short-MoCA <7). As shown in Table 3, in the univariate analysis, early PSCI survivors with a larger baseline hematoma and National Institutes of Health Stroke Scale (NIHSS) score, a dominant hemisphere or lobar ICH, and a higher MCV or mean corpuscular hemoglobin (MCH) appeared to have a lower ratio of cognitive recovery (*P* < 0.05). Even though the results of interest, like age and educational levels, barely meet criteria for significance at *P* < 0.05, we consider adjustments for multiple testing given the relatively small sample size in the present study. After selecting RBC indices through analysis of multicollinearity, our multivariate results showed a negative association between cognitive recovery and dominant-hemisphere hemorrhage [OR: 6.955 (1.604–30.162); *P* < 0.01], lobar ICH (OR: 8.363 (1.479–47.290); *P* = 0.016], educational years ≤ 9 [OR: 5.145 (1.254–21.105); *P* = 0.023], and especially altered RBC indices of high MCV levels [OR: 1.660 (1.171–2.354); *P* = 0.004] (Table 4).

**Table 3:** Prognostic factors in relation to follow-up cognitive improvement beyond the early post-intracerebral hemorrhage (ICH) cognitive impairment (*n* = 90).

	**Follow-up of early PSCI *n* = 90**	**Cognitive impairment *n* = 57**	**Cognitive recovery *n* = 33**	**Univariable analysis**		Multivariate analysis^*^	
				**OR and 95%CI**	***P*-value**	**OR and 95%CI**	***P*-value**
**Demographic variables**							
Age, years	68.70 ± 12.76	70.05 ± 13.06	66.36 ± 12.07	1.023 (0.989–1.059)	0.187	–	–
Male	58 (64.4%)	38 (66.7%)	20 (60.6%)	1.300 (0.534–3.163)	0.563		
Educational years ≤ 9	45 (50.0%)	30 (52.6%)	15 (45.5%)	1.333 (0.564–3.151)	0.512	5.145 (1.254–21.105)	**0.023**
Baseline NIHSS score	4 (2–6)	4 (2–7)	2 (2–5)	1.258 (1.054–1.500)	**0.011**	**–**	**–**
sBP on admission, mmHg	165.87 ± 25.04	165.47 ± 24.74	166.55 ± 25.92	0.998 (0.981–1.016)	0.844		
**Imaging variables**							
ICH volume, ml	3.99 (1.24–12.14)	6.43 (2.01–12.88)	2.39 (1.03–4.28)	1.082 (1.011–1.158)	**0.022**	**–**	**–**
Dominant-hemisphere hemorrhage	69 (76.7%)	48 (84.2%)	21 (63.6%)	3.048 (1.116–8.325)	**0.030**	6.955 (1.604–30.162)	**0.010**
Lobar hemorrhage	30 (33.3%)	27 (47.4%)	3 (9.1%)	9.000 (2.463–32.882)	**0.001**	8.363 (1.479–47.290)	**0.016**
Intracranial arterial stenosis	32 (35.6%)	19 (33.3%)	13 (39.4%)	0.699 (0.255–1.913)	0.486		
without assessment	24 (26.7%)	15 (26.3%)	9 (27.3%)				
White matter hyperintensities	53 (58.9%)	32 (56.1%)	21 (63.6%)	1.143 (0.232–5.632)	0.870		
without assessment	30 (33.3%)	21 (36.8%)	9 (27.3%)				
**Laboratory variables**							
CRP, mg/L	3.23 (3.02–5.50)	3.49 (3.02–10.14)	3.23 (3.02–4.24)	1.092 (0.987–1.208)	0.089		
RBC, × 10^12^/L	4.40 ± 0.56	4.26 ± 0.55	4.63 ± 0.51	0.273 (0.113–0.662)	**0.004**		
Hb, g/L	133.60 ± 17.67	132.00 ± 16.28	136.36 ± 19.79	0.986 (0.962–1.011)	0.259		
HCT, %	40.08 ± 4.70	39.82 ± 4.84	40.54 ± 4.48	0.967 (0.882–1.061)	0.482		
MCV, fL	91.52 ± 6.41	93.65 ± 4.92	87.86 ± 7.07	1.241 (1.091–1.413)	**0.001**	1.660 (1.171–2.354)	**0.004**
MCH, pg	30.51 ± 2.65	31.05 ± 1.67	29.56 ± 3.62	1.260 (1.041–1.524)	**0.018**	0.566 (0.293–1.092)	0.089
MCHC, g/L	332.93 ± 12.57	331.42 ± 6.24	335.55 ± 18.99	0.973 (0.939–1.009)	0.139		
RDW, %	13.31 ± 0.97	13.32 ± 0.88	13.30 ± 1.13	1.023 (0.656–1.595)	0.921		
PRO, g/L	65.51 ± 5.27	65.42 ± 6.07	65.66 ± 3.55	0.991 (0.913–1.076)	0.837		
ALT, U/L	16.39 ± 8.35	16.27 ± 8.61	16.60 ± 8.01	0.995 (0.945–1.048)	0.858		
AST, U/L	20.01 ± 4.33	20.39 ± 4.61	19.34 ± 3.76	1.060 (0.957–1.174)	0.267		
AST/ALT	1.46 ± 0.60	1.52 ± 0.65	1.34 ± 0.49	1.753 (0.796–3.861)	0.163		
Cr	68.82 ± 14.65	68.58 ± 16.70	69.22 ± 10.40	0.997 (0.968–1.027)	0.843		
eGFR, ml/min	87.64 ± 13.31	87.32 ± 14.25	88.19 ± 11.69	0.995 (0.963–1.028)	0.763		
FBG, mmol/L	6.04 ± 1.27	6.01 ± 1.35	6.10 ± 1.15	0.944 (0.675–1.321)	0.737		
LDL, mmol/L	2.31 ± 0.76	2.19 ± 0.75	2.51 ± 0.76	0.576 (0.323–1.027)	0.061		
HCY, μmol/L	13.68 ± 7.57	13.88 ± 9.34	13.34 ± 2.54	1.010 (0.951–1.073)	0.740		

*Values in bold indicate significant difference*.

* *Multivariate analysis adjusted for age, educational years, baseline NIHSS score, ICH volume, dominant-hemisphere hemorrhage, lobar hemorrhage, MCV, and MCH*.

**Table 4:** Logistic regression model in relation to follow-up cognitive recovery beyond the early PSCI (*n* = 90).

	***B***	**SE**	**Wald**	**OR**	**95%CI**	***P* value**
Educational years ≤ 9	1.638	0.720	5.175	5.145	1.254–21.105	0.023
Dominant-hemisphere hemorrhage	1.939	0.749	6.713	6.955	1.604–30.162	0.010
Lobar hemorrhage	2.124	0.884	5.773	8.363	1.479–47.290	0.016
MCV, fL	0.507	0.178	8.095	1.660	1.171–2.354	0.004
MCH, pg	−0.569	0.335	2.885	0.566	0.293–1.092	0.089
Constant	−30.908	8.811	12.306	<0.001	—	<0.001

* *Multivariate analysis adjusted for age, educational years, baseline NIHSS score, ICH volume, dominant-hemisphere hemorrhage, lobar hemorrhage, MCV, MCH*.

### The Baseline Cognitive Performances in Relation to Follow-Up Cognitive Improvement Beyond the Early PSCI

**Figure 1:**
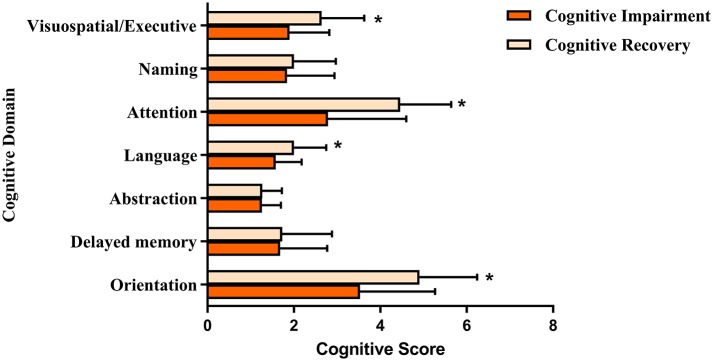
The baseline cognitive performance in relation to follow-up cognitive recovery beyond the early PSCL. ^*^P < 0.01 compared to the MoCA scroes of cognitive impairment group.

As shown in Figure 1, compared to those who had persisting cognitive impairment, ICH survivors who exhibited cognitive recovery at the follow-up were more likely to have better baseline cognitive performance in the domains of visuospatial/executive function (1.90 ± 0.92 vs. 2.64 ± 0.99; *P* = 0.001), attention (2.79 ± 1.81 vs. 4.46 ± 1.18; *P* < 0.001), language (1.58 ± 0.60 vs. 2.00 ± 0.75; *P* = 0.004), and orientation (3.53 ± 1.74 vs. 4.93 ± 1.33; *P* < 0.001). We did not observe significant differences in the domains of naming, abstraction, and delayed memory between the two groups of patients.

## Discussion

We conducted a longitudinal hospital-based study into prognostic factors associated with cognitive impairment and recovery after spontaneous ICH. We revealed a high incidence of early PSCI (75.2%), which was independently correlated with hemorrhage laterality, high sBP, and RBC indices. On the other hand, 36% of patients with early PSCI exhibited cognitive recovery at the 6-month follow-up, and this was closely associated with hematoma location, education, and MCV levels.

The incidence of post-ICH cognitive impairment reported in previous studies varies greatly, ranging from 19 to 77%. This may be a result of diversity in populations, cognitive evaluation tests, stage of stroke, severity of admission, regions, and other factors (19, 20). The first cross-sectional retrospective study that investigated the incidence of cognitive dysfunction in 78 ICH patients indicated that 77% of patients had cognitive impairment (21). However, it is hard to draw a strong conclusion from this given the major limitations of this study, namely, a mean cognitive evaluation period of 40 months, a small number of patients, and inclusion of patients with pre-existing cognitive impairment. Another prospective study that enrolled 218 ICH patients without preexisting dementia from the Prognosis of Intracerebral Hemorrhage cohort showed that 14.2% of patients developed new-onset cognitive impairment within 1 year, and this increased to 37% during the overall 4-year follow-up period (22, 23). These findings were in accordance with those of Biffi et al., who reported that 19% of ICH survivors developed dementia within 6 months in an observational prospective study of 738 patients (24). The contradictory findings of Moulin et al. and Biffi et al. may be related to differences in the timing of cognitive evaluation during the follow-up and the measure of cognition by the Mini-Mental State Examination (MMSE) and the Modified Telephone Interview for Cognitive Status test and not the MoCA. Given that that the MMSE is a rather crude evaluation of cognition and may lack sensitivity to vascular cognitive impairment, the MoCA has been currently recommended for the neuropsychological assessment of PSCI (14). Even though cognitive trajectories vary considerably, the rate of PSCI identified both in our results, and in previous studies is substantial (25), highlighting that this is a critical issue for clinicians, their ICH patients, and healthcare providers.

The rehabilitation process of stroke encompasses different stages, namely, the acute, postacute, and chronic stages. Cognitive functioning is typically observed to be affected in the acute stage (26), which refers to the immediate period of hospitalization in a stroke center to manage cerebrovascular disease. However, not all survivors would show constant cognitive dysfunction, which might recover to a certain extent during the postacute or chronic stages. However, the majority of studies lack data on baseline cognitive performance in the acute stage, limiting further investigation of prognostic factors for cognitive recovery. It should be noted that, due to the likelihood of confusion and delirium and perihematoma edema during the very-early stage of ICH, cognitive evaluations were mostly performed on the day of discharge in the present study (primarily 12–14 days after ICH onset). At this time, the ICH survivors had stable consciousness and condition, fulfilling the standard for transfer to a rehabilitation center. Moreover, for the majority of baseline NIHSS scores suggesting mild–moderate severity of admission, the validity of cognitive assessments results within 2 weeks appears to be believed. Our data corroborate a recent consecutive cohort study wherein 30% of patients who showed first-week cognitive impairment exhibited cognitive recovery after 6 months (7). Another longitudinal study (Cognition and Neocortical Volume after Stroke) demonstrated that visual memory and executive function were improved at 3 and 12 months after a stroke onset (9).

However, information about the potential prognostic factors of cognitive recovery in ICH survivors is limited. To the best of our knowledge, our study is the first to investigate for this issue, including demographic variables (age and educational level), laboratory tests (RBC indices and homocysteine), imaging characteristics (hematoma topography and volume, WMH, and larger artery stenosis), and vascular risk factors (diabetes and hypertension). The dominant hemisphere hemorrhage is more likely to acutely affect strategic domains, which consequently affect cognitive functions, as compared to non-dominant hematoma. Thus, the direct injury of the hematoma itself can partly explain the incidence of early PSCI. For survivors who showed cognitive recovery during follow-up, a recent hypothesis of strategic areas of the brain network predicting cognitive improvement might be reasonable (27). The pathological process of lobar ICH shows frequent association with cerebral amyloid angiopathy, which seems to closely correlate with Alzheimer's disease pathology (28). Therefore, a possibility of neurodegenerative processes could play a negative role in the cognitive recovery. However, we could not further address this question because we were unable to systematically determine the cause of cognitive dysfunction beyond the design of our study. Long educational history has been hypothesized to protect from brain injury through promoting brain reserve capacity (29). In addition, recent evidence further supports the hypothesis that cognitive reserve affects the cognitive recovery after ischemic stroke (30). Therefore, we assumed that this hypothesis might explain the cognitive recovery of ICH survivors with higher education during follow-up.

Of the RBC indices, MCV is the primary marker of variation in the size of the circulating RBCs. This indicator is a useful surrogate marker of systemic iron and/or folate status, and also reflects mitochondrial function (31, 32). In previous studies with a small number of hemorrhagic stroke survivors, abnormal RBC indices appeared to correlate with hematoma growth and poor functional outcomes (10, 33). However, these aforementioned studies were not designed to evaluate the effects of RBC indices on cognition after ICH, and the numbers of subjects included were too small to draw strong conclusions. In the present study, we not only recruited a larger number of ICH subjects but also provided more precise predictors by excluding likely confounding comorbid conditions such as preexisting dementia. In line with our published findings (12), we found that ICH survivors with high MCV levels were not only associated with early PSCI but also had a low chance to exhibit cognitive recovery.

Several mechanisms may explain the link between RBC indices and cognition in ICH survivors. First, increased MCV levels frequently reflect lower deformability of immature erythrocytes (34), which are more likely to rupture, consequently generating a substantial iron accumulation. Thus, the hypothesis of iron overload associated with incidence of cognitive impairment might be an appropriate explanation (35, 36). On the other hand, ICH survivors are prone to have decreases in cerebral blood flow in the perihematomal region, resulting in the presence of a hypoperfused penumbra (37–40). Although RBCs lack mitochondria, increased MCV levels may be a useful surrogate marker of subclinical mitochondrial dysfunction (41), suggesting a strong association with low oxygen consumption and altered metabolism in the perihematomal region (42). Last but not the least, the MCV level is one of the chronic parameters for measuring RBC, which is not normally affected in few hours of ICH. Thus, these changes may be due to a chronic nutrient-deficient state (e.g., vitamin B12, folate), which could be a result of aging. Homocysteine level, a useful indicator of folate metabolite, was similar across our groups, but our study was not designed to assess the vitamin B12 and folate levels.

Our data also indicated that ICH survivors with cognitive recovery were more likely to have better baseline cognitive performance in the domains of visuospatial/executive function, attention, orientation, and language. Given that these impaired domains are closely associated with characteristics of vascular cognitive impairment, rather than neurodegenerative disorders such as Alzheimer's disease, we assumed that either direct injury or secondary inflammatory processes induced by vascular etiology may be involved in the cognitive trajectory (43–45). Furthermore, our results are consistent with those from the Atherosclerosis Risk in Communities (ARIC) study, which reported the specific association of RBC indices with vascular cognitive impairment including processing speed and executive function (11). Thus, our finding of cognitive recovery from early PSCI supports and extends previous findings.

The main limitation in our study is the lack of mechanistic assessment to explain the role of RBC indices, like iron or vitamin B12 levels, which are not routinely tested in stroke treatments. Although the role of these indicators predicting cognition is controversial (46, 47), future studies should consider the possibility of these nutrition deficiencies as the potential culprits, as they seem to be associated with cognitive decline in all populations, not only ICH patients. Although we attempted to eliminate confounding effects of severe language impairment and unstable consciousness using the GCS scale, prior to proceeding with cognitive testing, another potential limitation could be that a dominant hemisphere lesion may raise concerns regarding the validity of our cognitive tests. Finally, regarding the small number of cases of cognitive recovery, we cannot exclude the risk of overfitting error in the logistic regression model. The main strengths of the present study include the prospective cohort of patients with ICH, and the cognitive assessments at two time points, both the acute stage and 6-month follow-up. We did not only investigated the prevalence of early cognitive impairment but also focused on the prognostic factors for cognitive recovery.

## Conclusion

This longitudinal study identified a high risk of incident cognitive impairment after an ICH. At the acute stage, within 2 weeks, our findings suggest a close association of cognitive decline with dominant-hemisphere hematoma as well as high sBP and MCV levels. However, during the 6-month follow-up, one in three early PSCI survivors exhibited cognitive recovery, and this recovery was related to dominant-hemisphere hematoma, lobar ICH, educational history, and MCV levels. To the best of our knowledge, this study is the first to explore prognostic factors of cognitive recovery beyond ICH onset, demonstrating hematoma location, and high MCV levels as the important predictors of cognitive performance after ICH, at both the acute and follow-up stages. The group of survivors exhibiting early PSCI will benefit from our study if future studies investigate the mechanisms underlying the role of these factors in cognitive recovery following ICH.

## Data Availability Statement

The datasets generated for this study are available on request to the corresponding author.

## Ethics Statement

The studies involving human participants were reviewed and approved by Ethics Committee of Shanghai Tenth People's Hospital (Shanghai, China). The patients/participants provided their written informed consent to participate in this study.

## Author Contributions

LG and YG conducted the study, analyzed the data, and both wrote the first draft of the paper. QY and HW both took part in participants enrollment, data collection, and cognitive evaluation. XZ and QD helped in follow-up and cognitive evaluation. RX, YZ, and XL all conceive the study, critically edited the manuscript and supervised the study.

## Conflict of Interest

The authors declare that the research was conducted in the absence of any commercial or financial relationships that could be construed as a potential conflict of interest.

## Abbreviations

ICH: intracerebral hemorrhage
NIHSS: National Institute of Health stroke scale
sBP: systolic blood pressure
CRP: C-reactive protein
RBC: red blood cell count
Hb: hemoglobin
HCT: hematocrit
MCV: mean corpuscular volume
MCH: mean corpuscular hemoglobin
MCHC: mean corpuscular hemoglobin concentration
RDW: red cell distribution width
PRO: total protein
ALB: albumin
ALT: alanine aminotransferase
AST: aspartate aminotransferase
AST/ALT: the ratio of aspartate aminotransferase to alanine aminotransferase
BUN: blood urea nitrogen
Cr: creatinine
eGFR: estimated glomerular filtration rate
FBG: fasting blood glucose
LDL: low-density lipoprotein
HCY: homocysteine.
